# Surface Roughness and Morphology Customization of Additive Manufactured Open Porous Ti6Al4V Structures

**DOI:** 10.3390/ma6104737

**Published:** 2013-10-22

**Authors:** Grzegorz Pyka, Greet Kerckhofs, Ioannis Papantoniou, Mathew Speirs, Jan Schrooten, Martine Wevers

**Affiliations:** 1 Department of Metallurgy and Materials Engineering, KU Leuven, Kasteelpark Arenberg 44 PB2450, Leuven B-3001, Belgium; E-Mails: greet.kerckhofs@mtm.kuleuven.be (G.K.); jan.schrooten@mtm.kuleuven.be (J.S.); martine.wevers@mtm.kuleuven.be (M.W.); 2 Prometheus, Division of Skeletal Tissue Engineering, KU Leuven, O&N 1, Herestraat 49 PB813, Leuven B-3000, Belgium; E-Mail: ioannis.papantoniou@med.kuleuven.be; 3 Biomechanics Research Unit, University of Liege, Liege B-4000, Belgium; 4 Skeletal Biology and Engineering Research Center: Laboratory for Tissue Engineering: Prometheus, KU Leuven, O&N 1, Herestraat 49 PB813, Leuven B-3000, Belgium; 5 Department of Mechanical Engineering, Division of Production Engineering, Machine Design and Automation, KU Leuven, Celestijnenlaan 300B, Leuven B-3001, Belgium; E-Mail: mathew.speirs@mech.kuleuven.be

**Keywords:** Ti6Al4V scaffolds, selective laser melting, surface roughness, surface modification, biomaterials

## Abstract

Additive manufacturing (AM) is a production method that enables the building of porous structures with a controlled geometry. However, there is a limited control over the final surface of the product. Hence, complementary surface engineering strategies are needed. In this work, design of experiments (DoE) was used to customize post AM surface treatment for 3D selective laser melted Ti6Al4V open porous structures for bone tissue engineering. A two-level three-factor full factorial design was employed to assess the individual and interactive effects of the surface treatment duration and the concentration of the chemical etching solution on the final surface roughness and beam thickness of the treated porous structures. It was observed that the concentration of the surface treatment solution was the most important factor influencing roughness reduction. The designed beam thickness decreased the effectiveness of the surface treatment. In this case study, the optimized processing conditions for AM production and the post-AM surface treatment were defined based on the DoE output and were validated experimentally. This allowed the production of customized 3D porous structures with controlled surface roughness and overall morphological properties, which can assist in more controlled evaluation of the effect of surface roughness on various functional properties.

## 1. Introduction

Porous structures hold unique physical properties (mechanical, thermal and electrical) that are related to their low density and architecture. These attributes open a wide variety of potential applications, such as insulation, packaging, filtering, medical implantology, as well as in the automobile, military shipping and aerospace industries [1,2,3,4,5]. At present, most porous structures still have a random morphology. The growing demand for porous structures with highly controlled architectural properties, coming from different industrial and scientific applications, has forced researchers to develop novel production techniques to enable the manufacturing of designed structures. A critical aspect in optimizing these production techniques and their post-production treatments is to control the morphological and mechanical properties from design up to the final manufactured functional structures [6,7]. Especially in the field of bone tissue engineering (TE), the tendency is to evolve from the use of random porous materials, like foams, to highly controllable microstructures that are based on a specific computer design. Controlled design improves the predictability of* in vitro* and* in vivo* experiments, as morphological parameters can be systematically varied, yielding better understanding of the role of morphological and mechanical effects [8]. Eventually, this knowledge may improve the probability of success of, for example, bone healing therapies.

Additive manufacturing (AM) is a state-of-the-art manufacturing method to build designed porous structures with a controlled geometry beneficial for orthopedic applications [6,7,9,10,11,12,13]. However, it is a bulk processing technology with limited control of final surface quality at the micro-scale [7,12,14,15]. Since the surface roughness influences the materials functional properties, such as the fluid dynamics [16,17], optical properties [18], frictional behavior [19], heat transfer [20], mechanical properties [21],* etc.*, the surface control requirements for AM products have considerably increased in recent years. Especially for biological applications, it is known that the interaction between the porous structure and the surrounding biological environment is strongly dependent on the surface properties [22,23]. Therefore, complementary and validated surface engineering strategies or post-AM surface treatments are needed. For instance, selective laser melting (SLM) of Ti-alloys into open porous structures intrinsically results in uncontrolled and inhomogeneous powder grain deposition on the beam surfaces [7,14,15]. It thus requires a suitable surface post-treatment to obtain controllable surface morphology, for example, when these open porous structures are used in orthopedic applications to support bone regeneration [24,25,26].

Controlled surface treatment of three-dimensional (3D) open porous structures is complicated, as it requires a process that uniformly treats the entire 3D structure, thus limiting the use of line-of-sight techniques, and that is tailored to the desired functional properties. Chemical processes offer this possibility, as acid-based solutions can penetrate into the porous structures through the interconnected pores and selectively dissolve the irregularities of the beam surfaces [7]. During chemical etching (CHE), chemical reactions occur at the interface between the substrate and the chemical solution, which leads to changes of the surface roughness [25,27]. In a previous study, the authors showed that CHE can be used to reduce the surface roughness of SLM-produced 3D Ti6Al4V open porous structures (SLM-Ti6Al4V) with a specific design [7]. However, further analysis revealed that the surface roughness of SLM produced 3D porous structures strongly depends on the initial design [28]. An increase in beam thickness resulted in larger amounts of attached powder particles, resulting in a higher surface roughness. For SLM, design of experiments (DoE) has been shown already to allow optimization of the production parameters, improving the density, geometrical characteristics [29,30] and surface roughness [31,32] of manufactured bulk objects. Therefore, in the present study, multi-factorial DoE was applied to provide an efficient procedure for controlled surface treatment of 3D porous structures. The main goal was to identify key factors influencing process outcomes, as well as their interactions [24,29,31,32,33,34]. As a proof-of-concept, DoE was applied as an SLM post-treatment case study with the aim to minimize the surface roughness of SLM-Ti6Al4V structures while obtaining predefined macro-morphological properties, the latter being similar to a non-processed reference material. Assessment on the effect of three process factors (designed beam thickness, chemical solution composition and treatment duration) on two output variables (final beam thickness and surface roughness) by systematically varying all factors within predefined ranges was carried out. The individual and combined effects of the process parameters were investigated.

## 2. Results

### 2.1. Ti6Al4V Open Porous Structures

**Figure 1:**
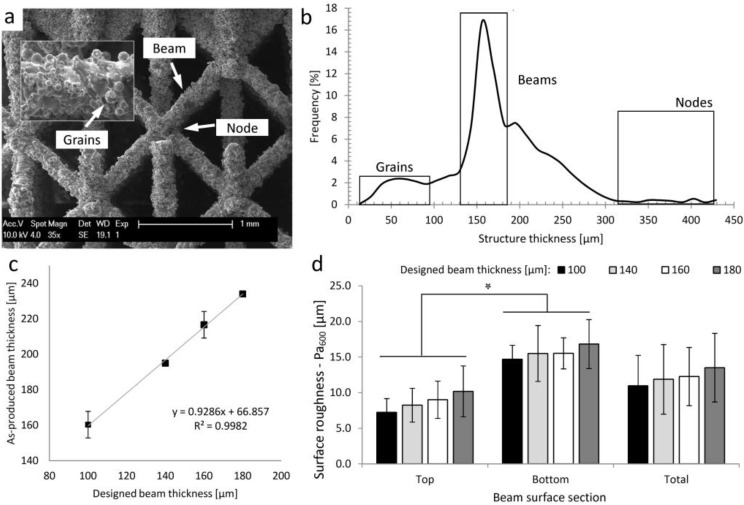
(**a**) A typical scanning electron microscopy (SEM) micrograph of a selective laser melting (SLM)-Ti6Al4V open porous structures with a designed beam thickness of 100 μm (s.100ap-ref), showing its complex morphology and (**b**) the structure thickness distribution of the s.100ap-ref (the left box in the distribution plot represents the grain thickness, the middle box, the beam thickness and the right box, the node thickness). The morphological parameters of the as-produced SLM-Ti6Al4V structures for the four designs, calculated using micro-CT images: (**c**) the designed and as-produced beam thickness and (**d**) the beam surface roughness, P_a600_.

Figure 1a presents typical scanning electron microscopy (SEM) micrographs of a reference material SLM-Ti6Al4V structure (further referred to as s.100ap-ref) with a designed beam thickness of 100 μm, showing its complex morphology. In Figure 1b, a typical structure thickness distribution of the s.100ap-ref structure is presented. The left peak in the distribution plot represents the thickness of the attached powder grains. The middle peak corresponds to the beam thickness and the right peak, to the node thickness. As shown in Figure 1c, there is an increase in the beam thickness of the as-produced SLM-Ti6Al4V structures compared to the designed thickness. The strong linear relationship (R^2^ = 0.99) between the designed and as-produced values indicated that there is a systematic off-set, thus allowing for a controlled post-treatment to compensate for it. The surface roughness analysis presented in Figure 1d showed a systematic increase in P_a600_ with increasing beam thickness. Additionally, a larger surface roughness was observed at the beam bottom sections for all designs.

### 2.2. Multi-Level Factorial Analysis for the Surface Treatment of SLM Porous Ti6Al4V Structures: Main and Interactive Effects

**Figure 2:**
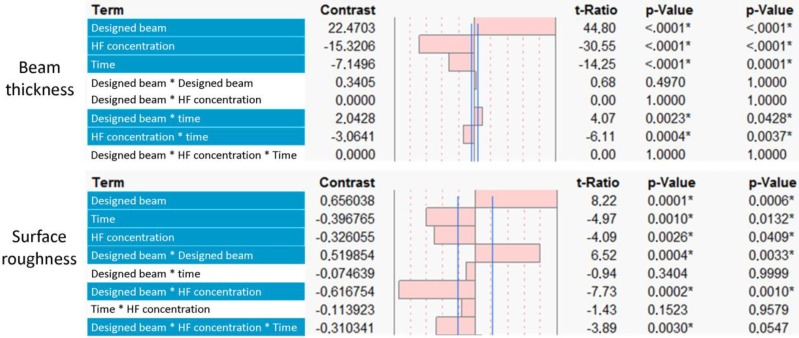
Multi-level factorial analysis of the main effects of the experimental factors [designed beam thickness, hydrofluoric acid (HF) concentration and surface treatment time] on the output variables (final beam thickness and surface roughness). The threshold of statistical significance is indicated by the vertical lines and was set at *p* < 0.1.

**Figure 3:**
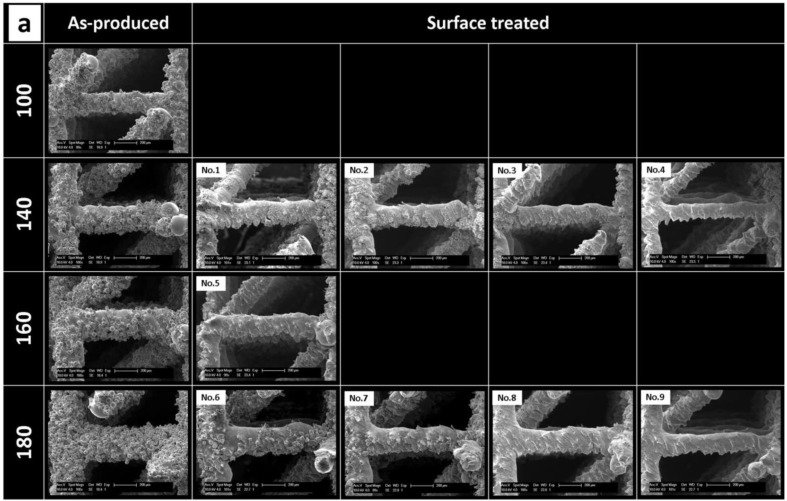

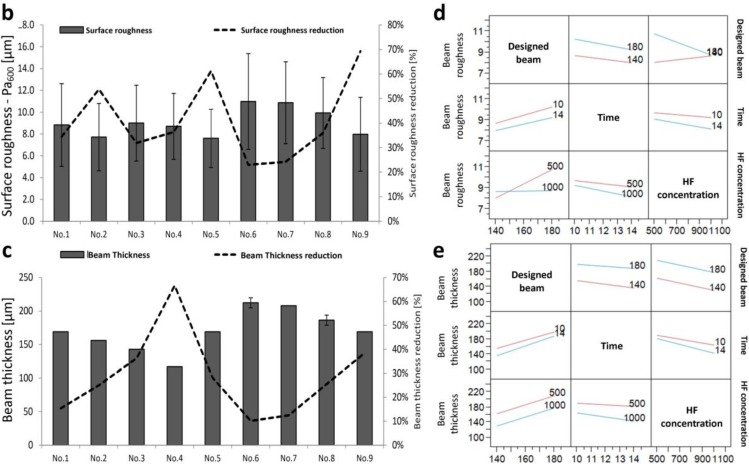
(**a**) Typical SEM micrographs of as-produced and surface-treated SLM-Ti6Al4V structures produced with a designed beam thickness of 100, 140, 160 and 180 µm, showing that (i) the amount of attached powder grains increased with increased beam thickness and (ii) the efficient removal of the attached particles depends on the designed beam thickness and surface treatment factors. Micro-CT-based quantitative analysis of the (**b**) surface roughness and (**c**) beam thickness of the surface-treated structures. The plot of the three factors influencing the (**d**) surface roughness and (**e**) beam thickness.

The designed beam thickness, hydrofluoric acid (HF) concentration and treatment duration were selected as factors influencing the two surface treatment process output variables,* i.e.*, final surface roughness and beam thickness. The positive and negative effects of these factors on the two output variables and their significance are shown in Figure 2. For both output variables, the designed beam thickness had the strongest effect. Concerning the CHE, the HF concentration had the most significant effect (*p*-values are shown in Figure 2) on the final beam thickness. For the reduction of the surface roughness, both the HF concentration and treatment time showed a significant influence. A combination of the HF concentration and the designed beam thickness had the strongest influence on the final surface roughness. Figure 3a presents SEM micrographs of a typical single beam prior to and after surface treatment for the four designs. The micro-CT-based surface roughness and beam thickness measurements of the as-produced and surface-treated structures are shown in Figure 3b,c. Figure 3d,e represents a two-factor linear interaction plot for all factors used in the experimental design, showing the significance of each process factor by the slope of the interaction line.

#### 2.2.1. Response to Surface Treatment: Surface Roughness Reduction

It can be seen (Figure 3a) that the low level values of both the surface treatment duration and the chemical solution concentration showed a limited removal of the powder particles (experimental run No. 1 and 6). An increase of the HF concentration resulted in a more effective removal of the attached particles (run No. 3 and 8), especially when combined with a longer treatment duration (run No. 4 and 9).

The surface roughness analysis (Figure 3b) showed, only for structures produced with a designed beam thickness of 180 μm, a progressive reduction in surface roughness with an increase of the treatment duration and HF concentration. In the case of structures with a designed beam thickness of 140 μm, the influence of the treatment parameters was less consistent. The largest change was observed for experimental run No. 2. Additionally, a higher reduction in surface roughness was found for structures with a designed beam thickness of 180 μm than for 140 μm when using the highest HF concentration and treatment duration (run No. 4 and 9). The interaction plots, presented in Figure 3d, confirmed the higher reduction of the surface roughness with increasing treatment duration and HF concentration.

#### 2.2.2. Response to Surface Treatment: Beam Thickness Reduction

Visual observation indicated a larger reduction in beam thickness for the high level values of the surface treatment parameters (Figure 3a). Relatively, for the same surface treatment parameters, the beam thickness was decreased more for structures produced with a designed beam thickness of 140 μm as compared to 180 μm ones (Figure 3b). For both beam thicknesses, the reduction rate increased with increasing treatment duration and HF concentration. The highest reduction in beam thickness was obtained for the high level value of the HF concentration and treatment duration. Interactions between the designed beam thickness-treatment duration and the designed beam thickness-HF concentration showed that the combined effect of these process factors influenced the beam thickness reduction. An opposite effect was observed between the designed beam thickness and the duration and HF concentration. Increasing the designed beam thickness reduced the beam thickness reduction caused by the surface treatment.

### 2.3. Case Study: Customized 3D Open Porous Structures

Based on the DoE predictions, the combined optimum process conditions required to produce the customized open porous structures were defined. Consequently, the Ti6Al4V alloy-based structures with a designed beam thickness of 140 μm (further referred to as s.140ap) were manufactured by SLM and chemically etched for 12.5 min using a solution with 0.6 wt% HF to obtain customized structures (further referred to as s.140custom) with a minimal surface roughness and a final beam thickness equal to the non-processed reference material (s.100ap-ref).

#### 2.3.1. Morphological Properties

**Figure 4:**
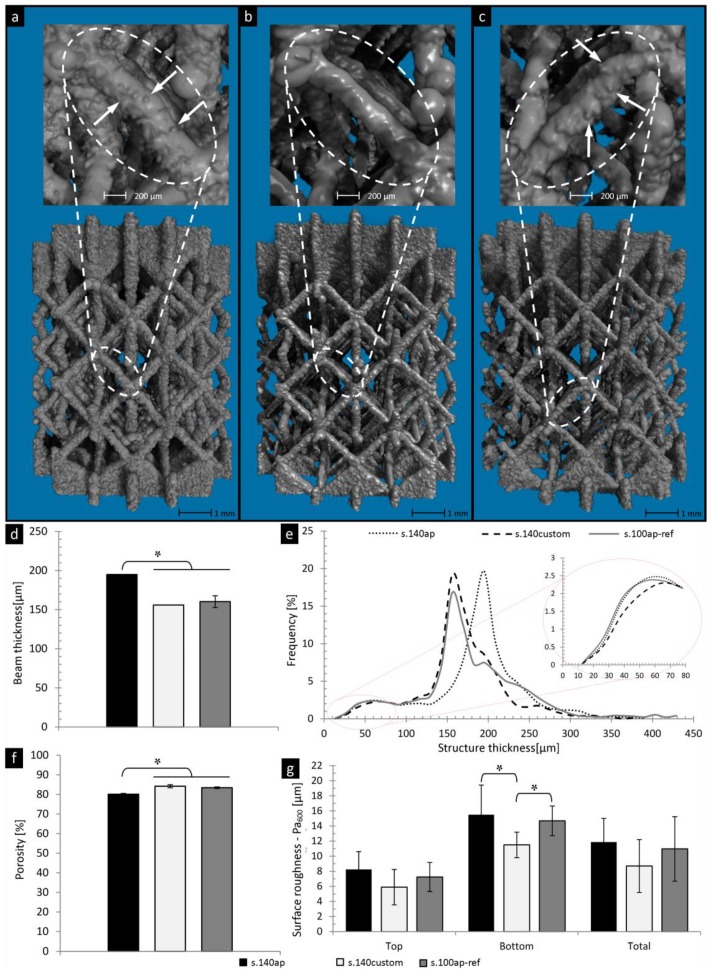
Micro-CT**-**based 3D visualization of the SLM-Ti6Al4V structures: (**a**) as-produced (s.140ap); (**b**) customized based on the design of experiments (DoE) outcome (s.140custom); and (**c**) reference material (s.100ap-ref), showing the changes of the surface roughness and beam thickness after surface treatment. Micro-CT-based quantitative analysis of the (**d**) beam thickness; (**e**) structure thickness distribution; (**f**) total porosity; and (**g**) surface roughness prior to and after surface treatment compared with the reference material.

Figure 4 presents the micro-CT 3D visualization and morphological analysis of the as-produced (s.140ap) and surface-treated (s.140custom) structures in comparison to the reference material (s.100ap-ref). 3D micro-CT based visual observation confirmed the presence of powder grains attached to the beam surface for structures s.140ap and s.100ap-ref (Figure 4a,c, respectively). DoE-based surface treatment of the s.140ap structures resulted in a reduced amount of powder grains and a smoother surface (Figure 4b). Micro-CT based morphological analysis indicated a significant difference between the beam thickness of the s.140ap and the reference material (s.100ap-ref), which was no longer the case after surface treatment (Figure 4d). Furthermore, no significant difference in total porosity between customized structures and the reference material was observed. Furthermore, Figure 4e shows good agreement between the structure thickness distribution of customized structures and that of the reference material. The main peak of the structure thickness distribution for the s.140ap (dotted line) was shifted to the left after surface treatment (dashed line) to overlap with the peak of the reference material (solid line). The removal of the particles attached to the beam surface because of the surface treatment also resulted in less objects with a thickness below 80 μm in comparison to the s.140ap structures and s.100ap-ref (detailed insert in Figure 4e). This was confirmed by the surface roughness analysis (Figure 4g), as a lower P_a600_ value was found after surface treatment in comparison to the reference material. Furthermore, the roughness of the beam bottom section of the customized structures was significantly different from that of the reference material.

#### 2.3.2. Mechanical Characterization

**Figure 5:**
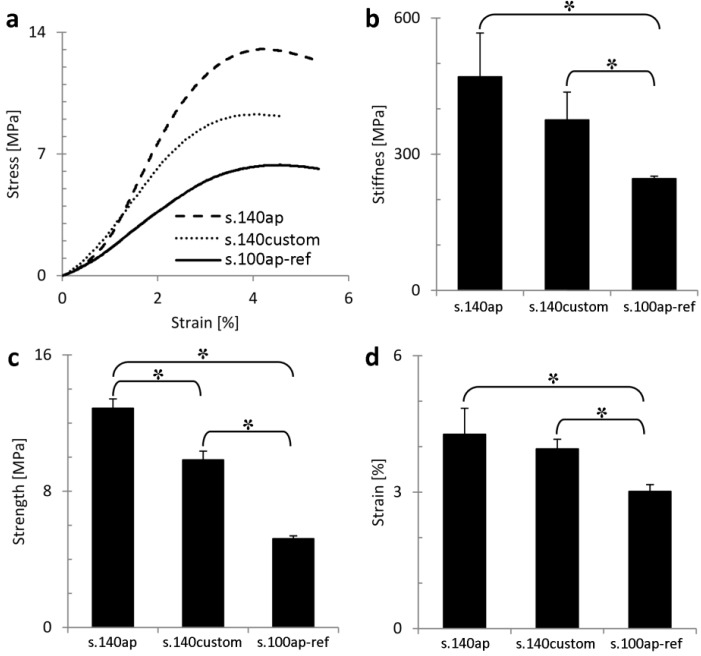
Mechanical analysis of the reference material (s.100ap-ref) and a structure with a designed beam thickness of 140 μm prior to (s.140ap) and after surface treatment (s.140custom): (**a**) typical stress-strain curves; (**b**) stiffness; (**c**) strength; and (**d**) ultimate strain.

Figure 5a shows typical stress/strain curves of the reference material (s.100ap-ref), the DoE-based as-produced (s.140ap) and customized (s.140custom) structures. Analysis of the stiffness (Figure 5b), strength (Figure 5c) and ultimate strain (Figure 5d) confirmed the differences in mechanical behavior observed in Figure 5a. The stiffness of the s.140ap was significantly higher than that of the reference material. A significant reduction in stiffness was observed after surface treatment; however, the stiffness of the customized structures was still significantly higher compared to the reference material. The same was observed for the ultimate compressive strength and strain. The difference in stiffness and strength between the s.140ap structures and the reference material was about 60% and 80%, respectively. The reduction in stiffness and strength after surface treatment was about 20% and 30%, respectively. The strength to weight ratio was 77.6, 78.5 and 44.2 [MPa/g] for structures s.140ap, s.140custom and s.100ap-ref, respectively.

## 3. Discussion

The general purpose of this study was to develop a strategy to produce, using SLM and post-production surface treatment, customized AM porous structures with a minimized and homogenous surface roughness and overall morphology. Apart from the macro-morphological properties, obtaining a desired surface quality for manufactured parts is one of the biggest SLM challenges. Previous studies [14,29,30,35] showed that SLM is a complex technique with a number of factors influencing the manufacturing process. Changing process parameters can introduce a non-linear response of the output products that can decrease the quality of manufactured objects [29,30,35]. There are several studies focusing on the optimization of the production parameters towards improvements of the surface quality of SLM manufactured objects [14,29,30]. It has been shown that the changing of scanning speed, the powder layer thickness, laser power [29,30], hatch distance [14], pulse duration, pulse frequency [32], production slope angle [15] or applying an additional laser re-melting step [36] significantly influences the surface quality of SLM manufactured parts. However, none of the proposed strategies allow production of 3D porous structures with a controlled surface roughness. Therefore, there is still a need for a robust surface post-production treatment strategy to obtain a controllable surface quality applicable for complex geometries.

Fukuda* et al.* [26] showed that chemical etching in combination with heat treatment improved the surface quality of Ti implants produced by SLM. Furthermore, specifically for SLM-produced 3D Ti6Al4V porous structures, Pyka* et al.* [7] developed a surface treatment protocol that enabled the removal of attached powder particles by chemical etching from the surface throughout the porous structures. However, one of the drawbacks of the post-treatment of SLM-produced structures is the additional post-production treatment step. Another limitation is the fact that the post-production surface treatment often significantly changes the geometry of the manufactured structure compared to its computed aided design (CAD) based definition [37]. Surface treatment by chemical etching leads to significant changes of the structure’s overall morphology and, thus, a reduction in mechanical properties [7]. Therefore, it is important to increase the controllability of the surface treatment process to limit additional manufacturing time and also to minimize the risk of further deviating from desired sample specifications. Additionally, a controlled surface treatment can compensate for the loss of morphological properties to ensure that customized structures fulfil the initial requirements for morphological and mechanical properties. Therefore, this protocol aimed at targeting a predefined surface roughness, while maintaining the desired morphological characteristics of 3D-Ti6Al4V porous structures [4] using a DoE strategy.

**Figure 6:**
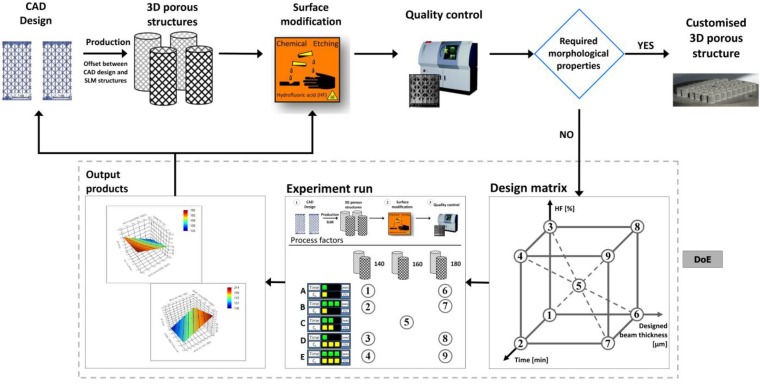
A schematic overview of the proposed production and surface treatment process for customized SLM-Ti6Al4V structures supported by the design of experiments (DoE) strategy.

DoE is an efficient tool that enables parallel investigation of multiple factors influencing a process. Multi-factorial analysis has been often used for process development in different applications [29,30,31,32]. A full factorial design and statistical Taguchi method were applied for SLM to optimize the production process parameters, such as laser speed, penetration depth and powder layer thickness [29,32]. In this study, it was shown that the reduction of surface roughness and the beam thickness of surface-treated 3D porous Ti6Al4V structures manufactured by SLM can be controlled by incorporating the DoE models into the manufacturing loop (Figure 6). In the first run, the influence of the process parameters on the output products was analyzed based on multi-factorial analysis. Subsequently, for the second run, customized 3D porous structures were produced based on the DoE model predictions.

**Figure 7:**
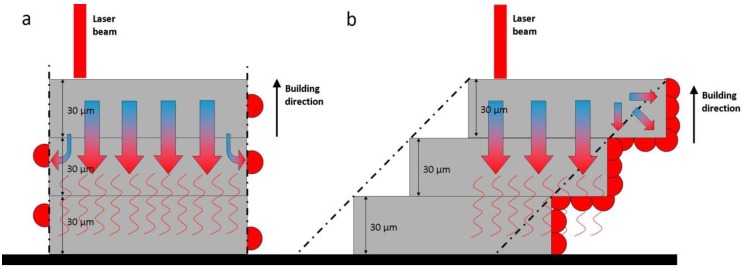
A visual illustration of heat flow and staircase effects for two different building angles. It highlights the increased amount of attached particles (red spheres) in relation to the building angle: (**a**) 90° building angle; (**b**) 45° building angle. The dash-dotted line corresponds to the designed beam contour.

For a robust AM-based production of 3D porous structures, customized by post-production surface treatment, thorough analysis of the interactions between initial structure properties and the subsequent surface treatment process conditions is crucial. For instance, in this study, a difference between designed and measured beam thickness was observed (Figure 1c). An increase of the as-produced beam thickness is intrinsically related to the limitations of SLM devices, but also to the fact that the process was carried out close to the technical device limits (*i.e.*, a melt pool larger than the laser spot size [6]). However, the linear relationship between designed and experimental values indicated that the design values could be considered as a stable input for production, as well as for the factorial design. Additionally, an increase in the designed beam thickness also resulted in a larger amount of attached powder particles. It was shown that there are two mechanisms that significantly increase the surface roughness of objects manufactured by SLM: (i) staircase formation and (ii) the attachment of the powder particles [15]. The staircase effect, caused by the stepped approximation by layers of curves and inclined surfaces of the manufactured object, is a common problem in AM processes influencing the surface roughness, but, also, the final structure thickness of the manufactured objects [6,15,38]. It has been shown that the real structure thickness increases while building objects at a larger slope [38]. In this study, the amount of attached powder particles was also larger in the case of structures produced with larger designed beam thickness. Additionally, the staircase effect enhances the powder particle attachment to the beam bottom section, which introduces the heterogeneity of the surface topology of the SLM manufactured objects [38]. In the SLM process, the introduced heat is mainly dissipated by conduction over the solid material. The lower the angle, however, the smaller the connected area to the solid material at the incline surface will be. Thus, for low building angles ≤45°, a greater number of partially molten powder particles are attached, due to heat dissipated by conduction. Part of the heat is conducted onto the powder bed, due to the building angle, causing adjacent particles to reach partial melting/sintering points and attach to the component surface, as shown by Figure 7 [15,38]. This implies that the applied surface treatment should be specifically optimized for each design.

In this study, factorial analysis revealed that the concentration of the chemical etching solution and the treatment duration strongly influenced the roughness reduction. However, the low level of HF concentration and treatment duration showed a limited roughness reduction for structures with a larger beam thickness. An increased presence of attached powder particles in the case of the structures with a strut size of 140 and 180 μm required exposure to a more aggressive reagent or longer treatment duration in order to dissolve surface irregularities. By increasing the HF concentration and surface treatment duration, a complete particle removal for structures with a designed beam thickness of 140 and 180 μm was obtained (Figure 2a, run No. 4 and 9), while showing comparable P_a600_ values (8.7 and 8.0 μm, respectively). A higher reduction in surface roughness was found for structures with a designed beam thickness of 180 μm than for the 140 μm case when using the highest HF concentration and treatment duration (run No. 4 and 9). This was due to the lower initial surface roughness of structures with a designed beam thickness of 140 μm (Figure 1d) compared to 180 μm. Despite complete particle removal, there was still a significant difference between the surface roughness at the beam top and bottom side, caused by the staircase effect [6,15,38]. The staircase effect introduced peaks in the beam bottom surface, which are clearly visible after particle removal (Figure 3a, run No. 3 and 8). Beam thickness reduction was also enhanced by increasing HF concentration. However, factorial analysis showed that an increase of the surface treatment parameters resulted in a similar beam thickness reduction for structures of 140 and 180 μm in contrast to the surface roughness. This latter phenomenon was related to the micro-CT-based beam thickness analysis, since the attached particles did not influence the beam size measurements. Therefore, the largest degree of change of the beam thickness for structures 140 and 180 µm was observed when all powder particles were removed (Figure 3b, run No. 3, 4, 8 and 9). Furthermore, it was observed that by increasing the designed beam thickness, a smaller reduction of the final surface roughness was obtained. Therefore, to achieve a complete removal of the attached particles and to significantly reduce the surface roughness, a higher level of the surface treatment conditions need to be applied. Based on the DoE output, it was concluded that the designed beam thickness and the CHE solution were the most significant factors influencing surface roughness and beam thickness reduction.

Despite the fact that as-produced Ti6Al4V open porous structures made by SLM are already used in different research [24,39,40] and commercial applications [41,42], it can be expected that an appropriate surface post-treatment would enhance the surface quality and, hence, improve the final functional outcome of these structures. As presented by Pyka* et al.* [43], a different cell behavior related to the surface morphology as the cells seeded on scaffolds with a rough surface (with attached powder particles) revealed a tendency to bridge places with high roughness, which hinder the cells from proliferation during 14 days of static culturing. In the case of smooth surface struts, a continuous cell monolayer was formed and covered the entire beam surface. Furthermore, the fluorescent images of the living cells taken from the side of scaffolds prior to and after surface roughness modification revealed a difference in the amount of the living cells attached to the untreated* versus* treated beam surfaces. The live-dead images did not show a significant amount of the dead cells, which implied the good biocompatibility of the Ti6Al4V scaffolds. It was concluded that the modification of the beam surface can be used to guide initial cell behavior seeded on 3D porous structures. However, a robust protocol for surface treatment allowing controlled surface roughness modification is required. Therefore, the main objective of this study was to obtain final structures with a minimized and homogenous surface roughness and predefined overall morphological properties that corresponded to a reference material. The prediction of DoE indicated that a beam thickness of 140 μm was the most optimal design to finally obtain structures with the desired morphology comparable to that of the reference material, since the 140 μm structure had the lowest initial surface roughness in comparison to the 160 or 180 μm structures. Due to the fact that the amount of attached particles for structures of 140 μm was lower, compared with,* i.e.*, the 180 μm structure, the low level of HF concentration and treatment duration were sufficient to ensure complete removal of the surface roughness. Additionally, the surface treatment parameters were selected accordingly to ensure the desired surface improvement in the minimal processing time, while maintaining the desired beam thickness. The overall good agreement between the predicted and actual morphological properties of customized structures implies a high reliability of the DoE model predictions for a controlled surface treatment of 3D T6Al4V porous structures. The overall morphological properties of the customized structures remained unchanged, while the surface quality was improved. Thus, this study showed that by incorporating DoE into the manufacturing and surface treatment processes, an improved controllability of the surface topology, but, also, the overall morphological properties of SLM-produced titanium implants, may be obtained across a production batch. In this way, surface roughness inhomogeneities caused by attached particles can be effectively eliminated for the reproducibility of cell proliferation, across structure batches during* in vitro* experiments. Furthermore, by chemical etching, a nano- and micro-pit-like topology can be created, which has been seen to also enhance cell attachment and proliferation [44].

Apart from biocompatible morphological properties, implant materials are expected to possess optimal mechanical properties. Two side effects of the post-production surface treatment were the reduction in average beam thickness and the increase in average pore thickness, which led to a change in the basic unit cell architecture [7]. It was shown that by changing the unit cell size and aspect ratio, the mechanical behavior of porous structures under compressive loading was influenced [6,7,45,46]. Therefore, a significant decrease in stiffness and strength was observed in the case of s.140custom structures, due to the post-production surface treatment (Figure 5). According to Van Bael* et al.* [7], additional material volume introduced by powder grains contributes little to the mechanical strength. However, in this study, the mechanical properties of customized structures were still significantly higher in comparison with the reference material, despite the fact that beam thickness and porosity were similar, while possessing a significantly reduced surface roughness. This was confirmed by the strength-to-weight ratio, which was also significantly larger for surface-treated structures. This implies that the surface roughness caused by the staircase effect might affect the mechanical behavior of porous structures produced by SLM. During surface treatment, both the surface roughness caused by the attached powder particles, as well as the staircase effect were reduced. The final product consisted of beams with a more homogenous thickness and surface quality, which could be a reason why the mechanical properties were greater in comparison with the reference material. Additionally, the mechanical properties of the customized structures could also be influenced by the SLM scan strategy, which was different in case of s.140ap structures in comparison to s.100ap-ref structures. In the case of s.100ap-ref structures, only a scan patch following the beam contour was applied. For s.140ap, a filling scan was applied followed by a contour scan. This two-step scan strategy was introduced for those structures to ensure complete melting of powder particles within the beam section defined in the design. This can lead to more uniform heat dissipation, which could result in a lower level of the internal stresses and, thus, higher mechanical properties [47]. Therefore, the influence of the local mechanical properties on the overall mechanical behavior and failure of 3D porous structures needs to be further explored.

## 4. Experimental Section

### 4.1. 3D Ti6Al4V Porous Structures

SLM was used to produce cylindrical 3D Ti6Al4V open porous structures starting from Ti6Al4V powder (Concept Laser GmbH, Lichtenfels, Germany). The powder was spherical, with diameters ranging from 25 to 45 μm. A non-commercial SLM machine was used, equipped with an IPG Yb:YAG fiber laser with a beam spot size of 80 μm. Cylindrical porous structures were designed using Magics software (Materialise NV, Haasrode, Belgium) to have an open porous unit cell. Four different architectures with a designed beam thickness of 100, 140, 160 and 180 μm and a designed pore thickness of 1 mm were used. Porous structures with a designed beam size of 100 μm were selected as the reference material (s.100ap-ref). The designed diameter and height of the porous structures were both 6 mm. More information about the porous structure design and production can be found in van Bael* et al.* [6]*.*

### 4.2. Surface Treatment

After SLM, the 3D Ti6Al4V porous structures were cleaned for 10 min in an ultrasonic bath in demineralized water, flushed with ethanol and air-dried to remove loose surface impurities and remaining powder particles from the pores. Next, CHE was performed by immersing the samples in a chemical solution based on hydrofluoric acid (HF), according to the protocol proposed by Pyka* et al.* [7]. The concentration of the HF solution ranged between 0.5 wt % and 1.1 wt %, and the duration of the surface treatment was between 10 and 14 min. Finally, the porous structures were rinsed with demineralized water, then with ethanol and, finally, air-dried.

### 4.3. Morphological Characterization

Microfocus X-ray computed tomography (micro-CT)-based morphological characterization of the 3D Ti6Al4V open porous structures, prior to and after surface treatment, was performed using a Phoenix NanoTom S (GE Measurement and Control Solutions, Wunstorf, Germany) equipped with a 180 kV/15 W high-performance nanofocus X-ray tube and a 2304 × 2304 pixel Hamamatsu detector. A tungsten target was used, and the applied voltage and current was 90 kV and 240 μA, respectively. A 0.3 mm cupper filter was installed. The exposure time was 500 ms, and a frame averaging of 1 and an image skip of 0 was applied, resulting in a total sample scan time of 20 min. After scanning, the radiographs were reconstructed using Phoenix datos|x 2.0 reconstruction software (GE Measurement and Control Solutions, Wunstorf, Germany). The resulting images had an isotropic voxel size of 6.5 μm. CTAn software (Bruker micro-CT, Kontich, Belgium) was applied for 3D morphological analysis of the micro-CT data. Automatic Otsu segmentation [48] was applied for binarization of the reconstructed micro-CT images. The total porosity, structure thickness and beam thickness were calculated for the as-produced and surface-treated samples. The structure thickness was calculated in 3D using a sphere-fitting algorithm [49]. The beam thickness was defined as the maximum of the structure thickness distribution.

### 4.4. Surface Roughness Analysis

Visual inspection of a single beam was done by scanning electron microscopy (SEM-Philips XL30 FEG, Eindhoven, The Netherlands) for qualitative analysis of the effectiveness of the applied surface treatment. Quantification of the beam surface roughness, which, according to the ISO 4287:1997 terminology, encompasses roughness, waviness and the unfiltered profile [50,51,52], was performed by a micro-CT-based protocol described by Kerckhofs* et al.* [53]. The profile lines of the beam surface extracted from 2D cross-sectional micro-CT images were used to calculate the arithmetic mean deviation of the surface roughness from its mean profile.

As for an arithmetic mean deviation of the surface roughness from its mean profile between 2 and 10 μm, the evaluation length required for correct analysis should be 12.5 mm, according to the ISO4288:1996 standards [52]. As for the porous structures investigated in this study, the average profile length available for measurement was only about 600 μm. We applied Equation (1) to quantify the beam surface roughness (P_a600_). The results should be considered relative to the length scale used.

(1) $$P_{a600}=\frac{1}{n}\sum_{i=1}^{n}|y_{i}|$$

with *n* corresponding to the number of data points in the *x* direction and *y*, the surface height relative to the mean line.

The beam surface roughness was quantified at the top and bottom of the beams prior to and after surface treatment.

### 4.5. Multi-Level Factorial Analysis

Based on previous findings [7,53], three process parameters termed as factors were used in the DoE to find their influence on the surface modification process of 3D Ti6Al4V porous structures: (i) designed beam thickness, (ii) surface treatment duration and (iii) concentration of HF in the CHE solution. The orthogonal array of the experimental design consisted of nine different conditions. Two significantly different levels were selected for each factor. For the designed beam thickness, the low level value was determined as 140 μm, while the high level value was 180 μm. The surface treatment duration was 10 min, as the low value, and 14 min, as the high level. The levels of the HF concentration were set at 0.5 wt % and 1.1 wt %. Experiments were performed in triplicate to improve the precision of the analysis. An overview of the experimental conditions and studied variables is shown in Table 1.

**Table 1:** An overview of the experimental conditions used for the three-factor two-level full factorial.

Experimental run	Factor A: Beam thickness	Factor B: Treatment time	Factor C: HF concentration (Cp)
	Low level (−): 140 μm	Low level (−): 10 min	Low level (−): 0.5%
	High level (+): 180 μm	High level (+): 14 min	High level (+): 1.1%
No. 1	−	−	−
No. 2	−	+	−
No. 3	−	−	+
No. 4	−	+	+
No. 5	center point	center point	center point
No. 6	+	−	−
No. 7	+	+	−
No. 8	+	−	+
No. 9	+	+	+

A statistical software package (JMP v.10, SAS) was used to detect the main effect of each factor on the output variables, namely, the final beam thickness and surface roughness, and to indicate the factors that would influence the surface treatment the most. The experiments were run in random order to minimize the bias in the observed responses. Because several factors are involved, multivariate analysis of variance was used to test the significance of each term in the equation and the goodness of fit of the regression model. The design matrix used for this experimental work is shown in the production process scheme (Figure 6). A linear model parameter estimation was performed by a least squares estimation. The main effects are included in the statistical model as follows:

(2) $$Y=b_{0}\sum_{i=1}^{n}b_{i}X_{i}+\sum_{i=1}^{n}b_{ij}X_{i}X_{j}+e$$

with *Y*, the predicted response; *b*, the parameter estimate; *X*, the coded value of the factor levels; and *e*, the residual error.

Statistical models were accepted when there was no lack of fit, no correlation in the residual plots and the residuals were normally distributed.

### 4.6. Case Study: Customization of Surface Roughness and Overall Morphology; and Compression Testing of SLM Open Porous Ti6Al4V Structures

The goal of the case study was to produce customized porous structures based on the DoE output parameters with a predefined beam thickness,* i.e.*, equal to the reference material, but with a minimized surface roughness. As presented in Figure 1c,d, the as-produced beam thickness of the reference material (s.100ap-ref) was 160 ± 7.5 μm, and the P_a600_ was 7.2 ± 1.9 μm and 14.7 ± 2 μm at the beam top and bottom surface, respectively. The surface roughness and final beam thickness of the customized structures, quantified as described above, will be compared to the ones of the reference material.

Additionally, to evaluate the influence of surface treatment on the mechanical behavior of customized structures, the mechanical properties of as-produced and surface-treated Ti6Al4V porous structures were determined by static compression testing. For compression testing, an in-house developed* in situ* loading stage equipped with a load cell of 3 kN was used at a constant compression rate of 0.2 mm/min. The stress-strain curves were used to quantify the stiffness, strength and ultimate strain of the as-produced and surface-treated porous structures. Since the curves did not show a distinct linear part in their stress-strain curve and its derivative did not show a plateau, the maximum slope of the stress-strain curve was regarded as the sample stiffness, in accordance with American Society for Testing and Materials (ASTM) standards E111 and D695 [6].

### 4.7. Statistical Analysis

A one-way ANOVA test was used to analyze batch variations using Analyze-it version 2.25. All values were reported as the mean ± standard deviation. For all results, *p*-values were determined and considered not significant if larger than 0.05.

## 5. Conclusions

In this study, it was found that surface treatment of 3D Ti6Al4V porous structures manufactured by SLM can be optimized by applying a design of experiments strategy. Multi-factorial experiments were used to explore the key factors that influence the robustness and controllability of a post-production surface treatment. It was observed that the concentration of the chemical etching solution enhanced the effectiveness of the surface treatment the most. Additionally, the DoE approach showed that the effectiveness of the surface treatment was also influenced by a combination of process factors, such as HF concentration and surface treatment duration. The initial beam thickness had a strong effect on the reduction of the surface roughness and final beam thickness. Based on the DoE output, optimal process conditions for production (SLM and surface treatment) of customized porous structures with a predefined surface roughness and morphological characteristics could be defined, showing the proof-of-concept of the DoE approach. Thus, this work showed that DoE is a useful tool to produce 3D porous SLM Ti6Al4V structures with a targeted morphology and surface roughness, which had a positive effect on the functional properties. Finally, modification of the beam surface can be used for controlling the cell behavior seeded on 3D porous structures. In that way, the most optimal surface properties for future designs and production of 3D scaffolds for TE can be looked for and validated experimentally. However, further experiments on as-produced and the 3D open porous Ti6Al4V structure customized in this study need to be performed.
